# Internalized Stigma and Associated Factors among Patients with Major Depressive Disorder at the Outpatient Department of Amanuel Mental Specialized Hospital, Addis Ababa, Ethiopia, 2019: A Cross-Sectional Study

**DOI:** 10.1155/2020/7369542

**Published:** 2020-08-17

**Authors:** Yadeta Alemayehu, Demeke Demilew, Getachew Asfaw, Henock Asfaw, Nigus Alemnew, Agitu Tadesse

**Affiliations:** ^1^Mettu University, Mettu, Ethiopia; ^2^Department of Psychiatry at University of Gondar, Gondar, Ethiopia; ^3^Ministry of Health, Addis Ababa, Ethiopia; ^4^Haramaya University, Dire Dawa, Ethiopia; ^5^Debre Berhan University, Debre Berhan, Ethiopia

## Abstract

**Background:**

Internalized stigma has been found to be widespread among patients with major depressive disorder. When internalized stigma exists in patients with depression at a high level, it worsens the treatment outcome and quality of life. So the aim of the study is to assess the magnitude of internalized stigma and associated factors among outpatients with major depressive disorder at Amanuel Mental Specialized Hospital, Addis Ababa, Ethiopia.

**Methods and Materials:**

An institutional-based cross-sectional study was conducted among 415 respondents from May 6 to June 13, 2019. Internalized stigma was assessed by using the internalized stigma of mental illness scale. Data was entered to Epi-data version 3.1 and analyzed using SPSS version 20. Bivariable and multivariable binary logistic analysis was done, and *p* values less than 0.05 were considered statistically significant with 95% CI.

**Results:**

The prevalence of high internalized stigma among patients with major depressive disorder was 33.5% (95% CI: 29.2, 38.3). Being single (AOR = 2.54, 95% CI: 1.30, 4.95), having an illness greater than or equal to 2 years of duration (AOR = 3.21, 95% CI: 1.66, 6.19), history of suicidal attempt (AOR = 2.33, 95% CI: 1.35, 3.99), nonadherence to treatment (AOR = 2.93, 95% CI: 1.62, 5.29), poor social support (AOR = 4.72, 95% CI: 2.09, 10.64), and poor quality of life (AOR = 3.16, 95% CI: 1.82, 5.49) were significantly associated with high internalized stigma at *p* value < 0.05.

**Conclusion:**

The magnitude of internalized stigma was high among patients with major depressive disorder. Reduction of internalized stigma through antistigma campaigns and supports given to patients at the earliest possible time is important to improve treatment outcome and quality of life and minimize suicidal behavior in patients with major depressive disorder.

## 1. Introduction

Stigma is “a mark of shame, disgrace or disapproval which results in an individual being rejected, discriminated against, and excluded from participating in a number of different areas of society” [1]. Stigma comprises problems of knowledge, attitude, and behavior. Stigma can also be categorized as public stigma (or social stigma) and personal experiences of stigma, which is measured in those patients with mental illness, including perceived, experienced, and internalized stigma [2–4].

Internalized stigma which is also termed self-stigma refers to the process by which individuals with mental illness apply negative stereotypes to themselves, expect to be rejected by others, and feel alienated from society [5, 6]. Internalized stigma usually occurs through three steps; first, a person with an undesired condition is aware of public stigma about their condition, then agree that these negative public stereotypes are true about the group, and subsequently, the person concurs that these stereotypes apply to him/herself. Internalized stigma can also exist without actual stigma from the public, which is more hidden and inside, so it seems to be the worst form of stigma against people with mental illness and can directly affect the patients' overall well-being [7].

Internalized stigma has been found to be widespread among patients with major depressive disorder. Accordingly, it is important that patients with depression are treated adequately. However, stigma appears to be a major roadblock in seeking treatment and continuing the treatment in the long run [8].

Globally, more than 300 million people of all ages suffer from depression according to the WHO report in 2017 [9]. Depression is the third leading contributor to the worldwide burden of diseases and predicted to become the second leading cause of the global disease burden by the year 2030 [10]. In Ethiopia, depression contributes to about 6.5% of the total burden of diseases [11].

When internalized stigma exists in patients with depression at a high level, it worsens: dysphoria, decline in self-esteem and quality of life (QoL), social avoidance or other forms of potentially maladaptive behavior, greater depression severity, delayed treatment response, nonadherence to treatment, and suicide [12, 13].

Internalized stigma is manifested by harm, decreased self-esteem, and increased depression. Patients feel shame and embarrassment about having a mental illness. These feelings limit social interactions and impair occupational functioning. When a patient labels himself/herself as a person in need of treatment, this may lead to further reduction of self-esteem, which constitutes the internalized stigma of seeking help. Thus, self-stigmatizing behavior is related more directly to experiencing mental illness and the other linked to seeking treatment [14].

Internalized stigma and discrimination is universally experienced by people with different mental disorders, and it remained a global public health concern over the years in both developing and developed countries even though reduction of stigma and discrimination has been taken as one of the core strategies of mental health global action programme (MH GAP) [15, 16].

Most studies done in western and Asian countries show that a high magnitude of internalized stigma among patients with depressive disorder ranges from 21.7 to 51.4% [17–23]. Accordingly, a study done in 13 European countries assessed the levels of internalized stigma among patients with mood disorders and they found that moderate to high levels of internalized stigma is present in 21.7% of individuals [4]. Studies done in Singapore shows that high levels of self-stigma in up to 51.4% of patients with depression [22].

Even though there are multifaceted impacts resulting from internalized stigma on patient, family, caregiver, and community worsening the treatment outcome and quality of life of patients with major depressive disorder, less concern is given to it. So this study was aimed at assessing the magnitude of internalized stigma and associated factors among patients with major depressive disorder.

## 2. Methods and Materials

An institution-based cross-sectional study was conducted from May 6 to June 13, 2019, at Amanuel Mental Specialized Hospital (AMSH). AMSH is the oldest and largest specialized mental health hospital in Ethiopia, located in the capital city, Addis Ababa. It provides care for patients coming from the entire nation of the country. The sample size was calculated by using a single population proportion formula and found to be 423. Systematic random sampling technique was used to select representative samples of patients with major depressive disorder based on DSM criteria, and those who had at least one follow-up visit at the outpatient department of Amanuel Mental Specialized Hospital were involved. Patients with major depressive disorder whose age ≥ 18 and on follow-up visit at Amanuel Mental Specialized Hospital during data collection period were included, and those who were unable to communicate were not included.

Data was collected by a face-to-face interview, correspondingly with reviewing the patient chart. Sociodemographic variables were collected by using a semistructured questionnaire.

The internalized stigma of mental illness (ISMI) scale was used for the assessment of internalized stigma. The ISMI scale is a widely used instrument consisting of 29 items, which have been grouped into five domains, subscales namely alienation, stereotype endorsement, social withdrawal, perceived discrimination, and stigma resistance. Except for stigma resistance domain, a higher score of the remaining four subscales indicates higher internalized stigma. The fifth subscale (stigma-resistance subscale) is conceptually different from the other parts of the scale and has lower internal consistency. So the 24-item 4-point Likert scale ISMI was used in the current study since it was validated and used in different studies in Ethiopia [24]. Each ISMI item contains a declarative statement about a potential stigma issue and participants respond to each statement by indicating their level of agreement: 1 = strongly disagree; 2 = disagree; 3 = agree; 4 = strongly agree [25]. In the current study, the internal consistency (Cronbach alpha) of overall ISMI is 0.92. For domains, the internal consistency for alienation domain is 0.84, the stereotype endorsement domain is 0.81, the discrimination experience domain is 0.90, and the social withdrawal domain is 0.77. Finally, a mean score of 1.00-2.50 was taken as the absence of high internalized stigma and a score of 2.51-4.00 was taken as the presence of high internalized stigma [25].

Medication adherence was assessed using the Medication Adherence Rating Scale. It has 10 “yes” or “no” questions. The patients are required to answer “no” for questions from 1 up to 6 and 9 up to 10, and “yes” for question 7 and 8 to become fully adherent. The total score of 6 or more indicates adherence and less than 6 indicates nonadherence [26].

Social support was measured by the Oslo social support scale which has a score ranging from 3 to 14 that will be interpreted as [3–8] is poor social support [9–11], is moderate social support, and [12–14] is strong social support [27].

The severity of depression was assessed by using an improved clinical global impression scale which has an objective CGI question that was filled by observing clinician and subjective CGI questions that were answered by participants. The question for both has seven responses which include normal, not at all ill, borderline mentally ill, mildly ill, moderately ill, markedly ill, severely ill, and among the most extremely ill patients which were recategorized as mild, moderate, and severe [28].

Quality of life was assessed using the EUROHIS-QOL 8-item index. It is prepared by the WHO and validated in patients with depressive disorder. It has eight questions that were answered by participants, and it was taken as poor quality of life if the average score level is <24 and good quality of life if the average score is ≥24 [29].

Suicidal attempt was asked whether the participant had felt so desperate that they had attempted suicide [30]. Duration of illness was categorized as if the duration of major depressive disorder was greater than or equal to 2 years, it was taken as chronic, and if less than 2 years, it was not chronic [31]. Income was categorized using the World Bank poverty line cut point for developing countries, and those who have an average monthly income of less than 1627 ETB (1.9$/day) taking 1$ = 28.54 ETB were taken as low income [32].

All questionnaires were translated into Amharic (local language) and back-translated into English to check its consistency. The questionnaires were pretested. Data collectors were trained on the questionnaires, and data was collected by psychiatric nurses. Data collectors were supervised daily, and the filled questionnaires were checked daily by the supervisor and principal investigator. Data was entered, cleaned, and stored by using EPI info version 3.5.1 and then exported into SPSS version 20 for analysis. Frequency and percentage were used to describe the data. Crude and adjusted OR was analyzed using logistic regression, the level of significance of association was determined at *p* value < 0.05, and the strength of the association was presented by adjusted odds ratio with 95% C.I.

### 2.1. Ethical Consideration

Ethical clearance was obtained from the joint ethical review committee of the University of Gondar and Amanuel Mental Specialized Hospital. A formal letter of permission was obtained from Amanuel Mental Specialized Hospital. The data collectors were clearly explained the aims of the study for study participants. The right was given to the study participants to refuse or discontinue participation at any time they want, and the chance to ask anything about the study was given. Data was collected after obtaining written consent from participants. Confidentiality was assured throughout the study period.

## 3. Results

A total of 415 study participants participated, giving a response rate of 98.1%.

### 3.1. Sociodemographic Characteristics of Respondents

The mean age of the respondents was 36 (±10.11 SD). Among participants, 220 (53%) were females and 187 (45.1%) were married (Table 1).

### 3.2. Clinical and Psychosocial Factors of the Respondents

The majority of respondents 292 (70.4%) had a duration of illness greater than or equal to 2 years. More than one-third of respondents 150 (36.1) have a history of admission for the illness, and 143 (34.5%) respondents have a lifetime history of suicidal attempt. About one-fourth of respondents 108 (26%) were nonadherent to their treatment. Most of the respondents had moderate social support 180 (43.4%), and almost half of the respondents 206 (49.6%) had poor quality of life (Table 2).

### 3.3. Magnitude of Internalized Stigma

**Figure 1:**
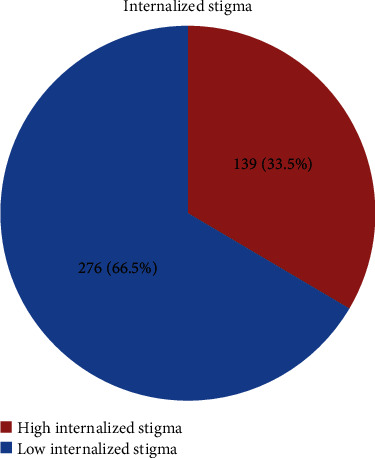
Magnitude of high internalized stigma among patients with major depressive disorder visiting adult outpatient department at Amanuel Mental Specialized Hospital, Addis Ababa, Ethiopia, 2019 (*n* = 415).

About 139 (33.5%) of respondents had high internalized stigma with (95% CI: 29.2, 38.3) having a raw and tabulated mean score of 56.39 ± 9.56 SD and 2.35 ± 0.40 SD, respectively (Figure 1).

**Figure 2:**
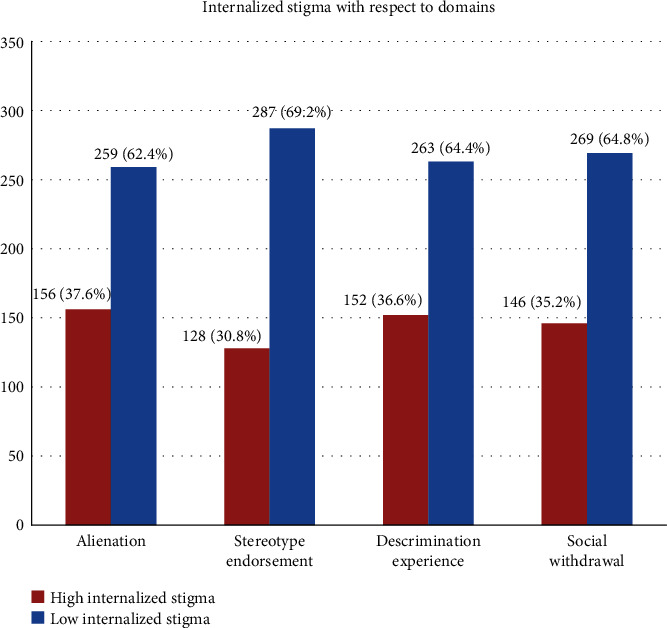
Magnitude of high internalized stigma with respect to domains among patients with major depressive disorder visiting adult outpatient department at Amanuel Mental Specialized Hospital, Addis Ababa, Ethiopia, 2019 (*n* = 415).

In domains, 156 (37.6%), 128 (30.8%), 152 (36.6%), and 146 (35.2%) respondents had a high internalized stigma score in alienation domain, stereotype endorsement domain, discrimination experience domain, and social withdrawal domain, respectively (Figure 2).

### 3.4. Factors Associated with High Internalized Stigma

In bivariable binary logistic regression variables, being female, single, widowed/divorced, jobless, lower income, duration of illness greater than or equal to 2 years, presence of comorbid physical illness, history of suicidal attempt, nonadherence to treatment, subjective severity of illness, objective severity of illness, poor social support, and poor quality of life were variables found to have a *p* value less than 0.2. These variables fulfilled minimum requirements for further multivariable binary logistic regression.

In multivariable binary logistic regression variables, being single, duration of illness greater than or equal to 2 years, history of suicidal attempt, nonadherence to treatment, poor social support, and poor quality of life were variables found to be a statistically significant association with high internalized stigma at a *p* value less than 0.05.

The odds of having high internalized stigma among respondents who were single were 2.54 times as compared to those who were married (AOR = 2.54, 95% CI: 1.30, 4.95).

The odds of having high internalized stigma among respondents who have a duration of illness greater than or equal to 2 years were 3.21 times higher as compared to those with a duration of illness less than 2 years (AOR = 3.21, 95% CI: 1.66, 6.19).

In this study, the odds of having high internalized stigma among respondents who have a lifetime history of suicidal attempt were 2.33 times higher than those without a history of suicidal ideation (AOR = 2.33, 95% CI: 1.35, 3.99).

The odds of having high internalized stigma among respondents who are not adherent to treatment were 2.93 times higher than those who are adherent to their treatment (AOR = 2.93, 95% CI: 1.62, 5.29).

The odds of having high internalized stigma among respondents who have poor social support were 4.72 times higher as compared to those who have strong social support (AOR = 4.72, 95% CI: 2.09, 10.64).

The odds of having high internalized stigma among respondents who have a poor quality of life were 3.16 times higher as compared to those who have a good quality of life (AOR = 3.16, 95% CI: 1.82, 5.49) (Table 3).

## 4. Discussion

The current study showed that the magnitude of high internalized stigma was 33.5% with 95% CI: 29.2, 38.3. The magnitude of this study was in line with the finding of different studies in India 34.1% [33] and USA 36.1% [34].

The magnitude was lower than studies done in India 41.1% [17] and Singapore 51.4% [22]. The possible reason for this discrepancy might be the difference in sample size and duration of treatment included; there are about 107 participants in the study in India [17] and 74 participants in the study in Singapore [22]. In the study done in Singapore, the data was collected from patients who have been on treatment for more than 1 year only so that the duration of being on illness might have an impact of increasing the magnitude of internalized stigma [22]. The difference in sociodemographic setting might be another reason for this variation.

However, the finding of the current study was higher than studies done in Nigeria 25.3% [35], Taiwan 25% [21], Israel 20% [23], and 13 European countries 21.7% [19]. The reason for the discrepancy might be due to differences in sample size, study design, data collection method, and tools. In the study done in Nigeria, the sample size (*n* = 116) [35] is different from the current study (*n* = 415). The study done in Taiwan used a self-stigma assessment tool which has only eight items, unlike ISMI which has twenty-four items. The other reason might be the study setting which is a general hospital in the study done in Taiwan [21]. The studies done in Israel was among old ages only [23]; since different study shows young age was among the factors associated with internalized stigma [17, 23], this might decrease the result. In addition, the mean age of participants in the current study is lower than most of the studies, indicating young age might influence the high magnitude of internalized stigma even though not significantly associated with the current study. In the study done in 13 European countries, mail survey and telephone interview were used [19], unlike the current study which is a face-to-face interview. The sociodemographic characteristics like income status and cultural difference might be another reason for the variation.

In this study, those who are single were 2.54 times more likely to have a high internalized stigma as compared to those who were married. This is supported by the study conducted in the USA [36]. The possible reason might be the sociocultural influence that separates them once their behavior was changed. The other reason might be those who are not married lack support which might be evidenced by poor social support which is significantly associated with the current study.

Having chronic major depressive disorder of ≥2-year duration was 3.21 times more likely to have a high internalized stigma than those who have less than 2 years of duration of illness. This is supported by different studies conducted in India [17] and Poland [20]. The possible reason for this association might be long-lasting poor social and occupational functioning resulting from the illness. Longer duration of illness might be associated with less controllability of depression weakening the hope for recovery from the illness and increasing idealization of the stigma [33].

Those who have a lifetime history of suicidal attempt were 2.33 times more likely to have high internalized stigma than those who do not have a suicidal attempt. This is supported by the study done in the Czech Republic [12]. The possible reason for the association might be unacceptability of suicidal attempt by sociocultural and religious ideologies. The other possible reason might be a higher presence of suicidal attempt among females than males [37] and a higher presence of internalized stigma among females than males [22]. Internalized stigma in these patients may also reversely increase the risk of suicidal attempt [13].

Those who were not adherent to their medication was 2.93 times more likely to have a high internalized stigma than those who were adherent to their treatment. This is supported by studies in the Czech Republic [27] and the UK [38]. The possible reason for the association might be those who were nonadherent to the treatment have poor controllability and recovery from the illness worsening their level of internalized stigma. The other possible reason might be the stigma that reversely decreases their follow-up visit limiting their relationship with others resulting in repetitive quitting of seeking the treatment.

Respondents having poor social support were 4.72 times more likely to have a high internalized stigma when compared with those who have strong social support. This association is supported by studies done in Nigeria [35], Singapore [22], Czech Republic [27], and the UK [38]. The possible reason for the association might be the introverting or internalizing of the social environment in which they might perceive the lack of a person who supports and helps them as isolation and stereotype due to their illness or against their illness. The other possible reason could also be explained by the fact that good social support increases an individual's sense of belongingness and integration into a group; it conversely decreases the feelings of alienation, discrimination, and social withdrawal which are critical parameters of internalized stigma.

Those with a poor quality of life were 3.16 times more likely to have a high internalized stigma than those who have a good quality of life. This finding is consistent with studies in Singapore [22], Czech Republic [39], and the UK [38]. The possible reason for this might be those with poor QoL tends to have poor socioeconomic life of weak social cohesion that might decrease an individual's sense of belongingness and integration into a group, resulting in an increment in the feelings of alienation, discrimination, and social withdrawal which are critical parameters of internalized stigma. The other possible reason for the association might be the internalized stigma that reversely separates them from the society and weakens the socioeconomic life of patients resulting in a poor quality of life.

The limitation of this study is that it cannot show a temporal causal relationship between internalized stigma and significant associated factors since a cross-sectional study design was utilized in this study.

## 5. Conclusion

The current study shows the magnitude of internalized stigma was high among patients with major depressive disorder. Those who have a high level of internalized stigma were shown significant association with poor social support, long duration of the illness, poor quality of life, nonadherence to treatment, history of suicidal attempt, and being single. Reduction of internalized stigma through antistigma campaigns and supports given to patients at the earliest possible time is important to improve treatment outcome and quality of life and minimize suicidal behavior in patients with major depressive disorder.

## Figures and Tables

**Table 1:** Sociodemographic characteristics of respondents with major depressive disorder visiting adult outpatient department at Amanuel Mental Specialized Hospital, Addis Ababa, Ethiopia, 2019 (*n* = 415).

Variable	Frequency (*N* = 415)	Percent (%)
Sex		
Male	195	47
Female	220	53
Age		
18-24	56	13.5
25-34	130	31.3
35-44	121	29.2
≥45	108	26
Religion		
Orthodox	227	54.7
Muslim	107	25.8
Protestant	81	19.5
Marital status		
Married	187	45.1
Single	136	32.8
Divorced/widowed	92	22.2
Occupation		
Employed	115	27.7
Merchant	78	18.8
Farming	57	13.7
Jobless	112	27
Others	53	12.8
Education status		
No formal education	70	16.9
Elementary	111	26.7
High school	111	26.7
College and above	123	29.6
Residency		
Urban	314	75.7
Rural	101	24.3
Average monthly income		
<1627 ETB (<57$)	278	67
≥1627 ETB (≥57$)	137	33

Others: students, housewives, and daily laborers.

**Table 2:** Description of clinical and psychosocial factors of the respondents with major depressive disorder visiting adult outpatient department at Amanuel Mental Specialized Hospital, Addis Ababa, Ethiopia, 2019 (*n* = 415).

Variables	Frequency	Percent (%)
Duration of illness		
<2 years	123	29.6
≥2 years	292	70.4
History of admission		
Yes	150	36.1
No	265	63.9
Comorbid physical illness		
Yes	105	25.3
No	310	74.7
Lifetime suicidal attempt		
Yes	143	34.5
No	272	65.5
Adherence to treatment		
Nonadherent	108	26
Adherent	307	74
Subjective severity of depression		
Mild	119	28.7
Moderate	246	59.3
Severe	50	12
Objective severity of depression		
Mild	146	35.2
Moderate	229	55.2
Severe	40	9.6
Social support		
Poor	166	40
Moderate	180	43.4
Strong	69	16.6
Quality of life		
Poor	206	49.6
Good	209	50.4

**Table 3:** Bivariable and multivariable binary logistic regression analysis showing an association between high internalized stigma and associated factors among patients with major depressive disorder visiting adult outpatient department at Amanuel Mental Specialized Hospital, Addis Ababa, Ethiopia, 2019 (*n* = 415).

Explanatory variables	High internalized stigma		COR (95% CI)	AOR (95% CI)	*p* value
	Yes	No			
Sex					
Female	91	129	2.16 (1.42, 3.29)	1.70 (0.96, 3.01)	
Male	48	147	1	1	
Marital status					
Married	40	147	1	1	
Single	61	75	2.99 (1.84, 4.86)	**2.54 (1.30, 4.95)**	**0.006**
Divorced/widowed	38	54	2.59 (1.50, 4.45)	1.57 (0.79, 3.10)	
Occupational status					
Employed	35	80	1	1	
Merchant	24	54	1.016 (0.54, 1.89)	1.07 (0.47, 2.45)	
Farmer	14	43	0.74 (0.36, 1.53)	1.08 (0.42, 2.80)	
Jobless	53	59	2.05 (1.19, 3.54)	1.11 (0.47, 2.59)	
Others	13	40	0.743 (0.35, 1.56)	0.70 (0.25, 1.98)	
Income					
<1627 ETB (<57$)	108	170	2.17 (1.36, 3.47)	1.06 (0.51, 2.20)	
≥1627 ETB (≥57$)	31	106	1	1	
Duration of illness					
<2 years	21	102	1	1	
≥2 years	118	174	3.29 (1.95, 5.57)	**3.21 (1.66, 6.19)**	**0.001**
Comorbid physical illness					
Yes	43	62	1.55 (1.01, 2.42)	1.18 (0.65, 2.14)	
No	96	214	1	1	
Suicidal attempt					
Yes	73	70	3.26 (2.12, 5)	**2.33 (1.35, 3.99)**	**0.002**
No	66	206	1	**1**	
Adherence to treatment					
Nonadherent	65	43	4.76 (2.98, 7.58)	**2.93 (1.62, 5.29)**	**<0.001**
Adherent	74	233	1	1	
Objective severity					
Mild	40	106	1	1	
Moderate	78	151	1.37 (0.86, 2.16)	1.20 (0.68, 2.14)	
Severe	21	19	2.93 (1.42, 6.01)	2.54 (0.95, 6.77)	
Social support					
Poor	91	75	5.23 (2.66, 10.28)	**4.72 (2.09, 10.64)**	**<0.001**
Moderate	35	145	1.04 (0.51, 2.11)	1.00 (0.43, 2.36)	
Strong	13	56	1	1	
Quality of life					
Poor	96	110	3.37 (2.19, 5.19)	**3.16 (1.82, 5.49)**	**<0.001**
Good	43	166	1	1	

Others: students, housewives, and daily laborers; chi‐square = 4.583; df = 8; Hosmer and Lemeshow test = 0.801.

## Data Availability

The data used to support the findings of this study are available from the corresponding author upon request.

## Abbreviations

AMSH: Amanuel Mental Specialized Hospital
AOR: Adjusted odds ratio
CGI: Clinical global impression scale
CI: Confidence interval
COR: Crude odds ratio
ETB: Ethiopian birr
EUROHIS QOL: European Health Interview Survey Quality of Life
IQR: Interquartile range
ISMI: Internalized stigma of mental illness
MARS: Medication adherence rating scale
MSc: Masters of science
OPD: Outpatient department
OR: Odds ratio
OSS: Oslo social support scale
QoL: Quality of life
SD: Standard deviation
SPSS: Statistical Package Software for Social Sciences
UK: United Kingdom
USA: United States of America
WHO: World Health Organization.
